# Personalised thrombo-embolic risk prediction after endometrial cancer surgery: an explainable AI approach using SHAP

**DOI:** 10.1038/s41746-026-02782-4

**Published:** 2026-05-27

**Authors:** Qing Zhou, Fudan Liu, Jiaxiu Li, Qiyin Zhou, Xiaorong Xiang, Cheng Dai, Lianli Hen, ShouLi Dao, Mingjie Zhang, Bowen Gao, Yuxi Li, Lin Xu, Yonghu Chang, Donghong Wang

**Affiliations:** 1 Department of Obstetrics and Gynecology, Affiliated Hospital of Zunyi Medical University, Zunyi, Guizhou China; 2 School of Management, Zunyi Medical University, Zunyi, Guizhou China; 3 Department of Obstetrics and Gynecology, Yanhe Tujia Autonomous County People’s Hospital, Yanhe, Guizhou China; 4 Department of Respiratory and Critical Care Medicine, Shanghai Changzheng Hospital, Naval Medical University, Shanghai, China; 5 Department of Obstetrics and Gynecology, Guizhou Provincial People’s Hospital, Guiyang, Guizhou China; 6 Department of Obstetrics and Gynecology, The Third Affiliated Hospital of Zunyi Medical University, Zunyi, Guizhou China; 7 Department of Obstetrics and Gynecology, Liupanshui Maternal and Child Health Hospital, Liupanshui, Guizhou China; 8 Key Laboratory of Cancer Prevention and Treatment of Guizhou Province, Zunyi, Guizhou China; 9 School of Medical Information Engineering, Zunyi Medical University, Zunyi, Guizhou China

**Keywords:** Cancer, Computational biology and bioinformatics, Diseases, Health care, Medical research, Risk factors

## Abstract

To address the clinical challenge of postoperative lower extremity deep vein thrombosis (LEDVT) in endometrial cancer care, this study establishes an explainable machine learning framework for personalized risk prediction. Utilizing perioperative data from 841 patients across multiple centers, we evaluated 26 machine learning algorithms combined with diverse data augmentation techniques. The Support Vector Machine (SVM) model emerged as the most robust architecture, refined through recursive feature elimination to a concise four-variable set comprising postoperative D-dimer, age, fibrinogen, and clinical stage. The model demonstrated superior discriminative performance, achieving an area under the curve (AUC) of 0.828 in internal validation and 0.819 in an independent external cohort. To bridge the gap between “black-box" AI and clinical trust, we integrated SHapley Additive exPlanations (SHAP) to quantify individual feature contributions, revealing non-linear associations such as the critical risk threshold for D-dimer levels. Finally, a web-based decision support interface was implemented to provide real-time, interpretable risk assessments. By combining high predictive accuracy with transparent decision logic, this approach offers a precise tool for identifying high-risk patients and optimizing postoperative management in endometrial cancer care.

## Introduction

Endometrial cancer(EC) is one of the common gynecological malignancies, and surgical intervention remains its primary treatment modality^1^. However, lower extremity deep venous thrombosis (LEDVT) represents a frequent and serious postoperative complication in EC patients^2,3^. Without timely intervention, dislodgement of the thrombus can lead to life-threatening pulmonary embolism, posing a significant risk to patient survival^2^. Therefore, in clinical practice, the early and accurate identification of high-risk patients for LEDVT is crucial for initiating timely prophylactic measures and improving patient prognosis^4^

Currently, general risk assessment tools such as the Caprini score and Wells score are widely used to predict venous thromboembolism^5^. However, the predictive accuracy of these tools is limited in the gynecological oncology population^6^. These tools are mostly static scoring systems that cannot incorporate postoperative dynamic indicators (e.g., D-dimer trends), nor do they adequately quantify the synergistic impact of tumor-specific factors (such as FIGO stage and histological grade) on thrombotic risk^7,8^. Consequently, their predictive efficacy is constrained, making it difficult to achieve individualized dynamic risk early warning. In recent years, ML methods derived from electronic medical records have gained increasing clinical attention in the field of postoperative complication prediction. ML can handle complex, high-dimensional nonlinear data, which makes it particularly suitable for predicting postoperative complications such as deep vein thrombosis that result from multifactorial interactions^9^. To date, studies in orthopedics, general surgery, and other fields have successfully applied ML models to predict the risk of deep vein thrombosis and demonstrated good performance^10^. However, in the specific patient population of EC, such research remains scarce^11^.

Although ML models offer strong predictive performance, their decision-making process is often regarded as a “black box," thereby undermining clinical trust and adoption^12^. To overcome this limitation, the SHapley Additive exPlanations (SHAP) framework has been proposed^13^. It can simultaneously provide both global feature importance rankings and local prediction explanations for individual patients, clearly delineating the direction and magnitude of each clinical variable’s contribution to the prediction outcome for a specific individual. Thereby, it transforms the “black box" into a trustworthy clinical decision-support tool^14^.

This study aimed to develop and validate an individualized prediction model for lower extremity deep venous thrombosis (LEDVT) after surgery in patients with EC, based on routinely available perioperative clinical data. The SHapley Additive exPlanations (SHAP) interpretable artificial intelligence method was systematically applied in this field for the first time. The goal was not only to develop a high-accuracy predictive tool but also to achieve both global and individualized explanations of the model’s decision-making logic. Thereby, it sought to provide clinicians with a transparent and trustworthy risk-assessment basis, promoting the evolution of postoperative LEDVT management toward precision and earlier intervention.

## Results

### Patient characteristics

**Fig. 1:**
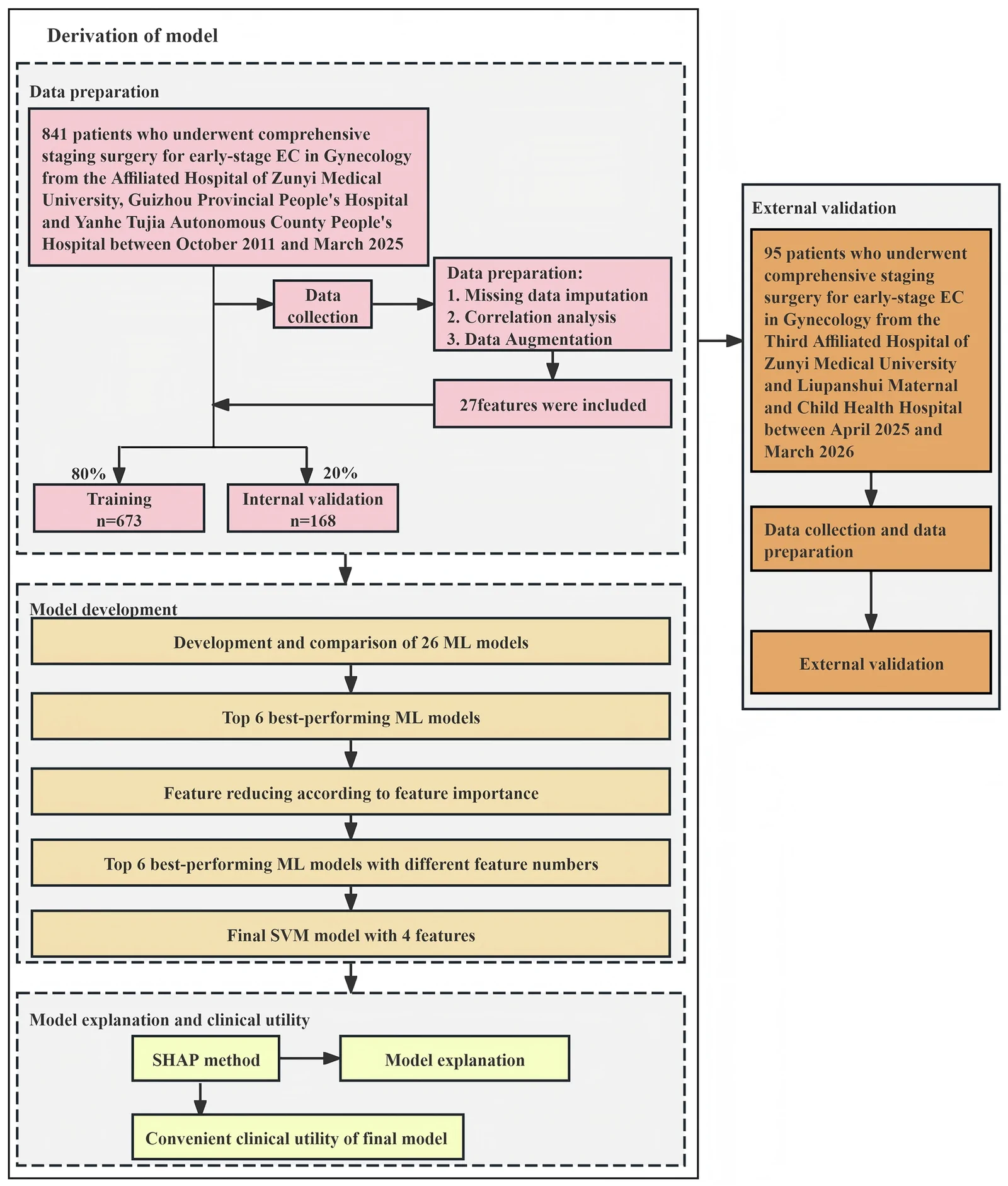
Flow chart of the study design and patient selection process. The figure delineates the comprehensive research methodology, encompassing the initial screening of 841 endometrial cancer patients, the strategic partitioning into training, internal validation, and external validation cohorts, and the subsequent phases of model development and performance evaluation. EC endometrial cancer, LEDVT lower extremity deep venous thrombosis, ML machine learning, SVM support vector machine, SHAP shapley additive explanation.

This retrospective study enrolled a total of 841 patients as the derivation cohort for predictive model development. During the study period, 841 consecutively screened patients were randomly divided in an 8:2 ratio into a training set (*n* = 673) and an internal validation set (*n* = 168), with an external validation cohort consisting of 95 patients used for external validation. The study flow is detailed in Fig. 1. Among the 841 patients who underwent radical surgery for endometrial carcinoma, 72 (8.6%) developed postoperative thrombosis. The baseline characteristics of the thrombosis and non-thrombosis groups are presented in Table 1. Compared with patients without thrombosis, those in the thrombosis group were older (mean age 56.0 years vs. 53.0 years), had a higher proportion of postmenopausal status (72.2% vs. 55.1%), and a greater prevalence of hypertension (34.7% vs. 24.2%). In terms of disease characteristics, the thrombosis group had a higher percent age of advanced clinical stage (stage II: 38.8% vs. 11.4%) and a greater incidence of lymphovascular invasion (26.4% vs. 8.3%) compared to the non-thrombosis group. Postoperative indicators showed that the thrombosis group had higher mean D-dimer levels (1.8 mg/L vs. 0.8 mg/L) and longer mean postoperative bed-rest time (48 h vs. 24 h). Additionally, the proportions of intraoperative blood transfusion, central venous catheter placement, and use of anticoagulant or hemostatic agents also differed between the two groups.

**Table 1:** Comparison of demographic and clinical characteristics and outcomes between non-LEDVT and LEDVT in the derivation cohort

Variables	Non-LEDVT (*n* = 769)	LEDVT (*n* = 72)	*P* value
BMI	24.4 [22.2–27.0]	24.2 [22.1–26.4]	0.518
Clinical stage(II), *n* (%)	88 (11.4%)	28 (38.8%)	<0.001
Thrombin time, s	18.1 [14.9–20.3]	17.1 [12.1–19.4]	0.095
Age, year	53.0 [48.0–57.0]	56.0 [50.8–62.0]	<0.001
Menopausal status, *n* (%)	6 (0.8%)	6 (8.3%)	<0.001
Central venous catheter placement, *n* (%)	519 (67.5%)	53 (73.6%)	0.287
Diabetes mellitus, *n* (%)	68 (8.8%)	15 (20.8%)	0.001
Menopausal status, *n* (%)	424 (55.1%)	52 (72.2%)	0.005
Vascular invasion, *n* (%)	64 (8.3%)	19 (26.4%)	<0.001
Anticoagulant administration, *n* (%)	364 (47.3%)	44 (61.1%)	0.025
Hemostatic agent administration, *n* (%)	224 (29.1%)	19 (26.4%)	0.624
Maximum tumor diameter, cm	2.0 [0.0–4.0]	3.0 [0.7–5.0]	0.012
Intraoperative blood loss, ml	80.0 [50.0–180.0]	100.0 [50.0–200.0]	0.023
Intraoperative fluid administration volume, ml	2300.0 [1600.0–3100.0]	2550.0 [1737.5–3125.0]	0.161
Intraoperative blood transfusion volume, ml	42.33 [0–2100]	130.56 [0–2000]	0.026
Postoperative D-dimer level, *μ*g/mL	0.8 [0.5–1.2]	1.8 [1.0–2.6]	<0.001
Postoperative massage of both lower limbs, *n* (%)	745 (96.9%)	55 (76.4%)	<0.001
Postoperative continuous bed rest time, h	24.0 [24.0–48.0]	48.0 [24.0–48.0]	<0.001
Activated partial thromboplastin time, s	27.1 [24.4–31.2]	27.4 [25.5–34.2]	0.027
Pathological grade(I), *n* (%)	534 (69.4%)	33 (45.8%)	<0.001
Total white blood cell count, 10^9^ L^−1^	8.3 [6.5–11.1]	8.6 [6.7–11.3]	0.786
Hematocrit, L/L	0.3 [0.3–0.4]	0.3 [0.3–0.3]	0.004
Total red blood cell count, 10^12^ L^−1^	3.8 [3.5–4.2]	3.7 [3.3–4.0]	0.009
Fibrinogen level, g/L	3.0 [2.4–4.1]	4.2 [3.1–5.3]	<0.001
Total platelet count, 10^9^ L^−1^	206.0 [170.0–255.0]	215.0 [163.8–258.0]	0.581
Hypertension, *n* (%)	186 (24.2%)	25 (34.7%)	0.049
Operation time, min	235.11 (43–689)	263.46 (95–475)	0.007

### Model development and performance comparison

**Fig. 2:**
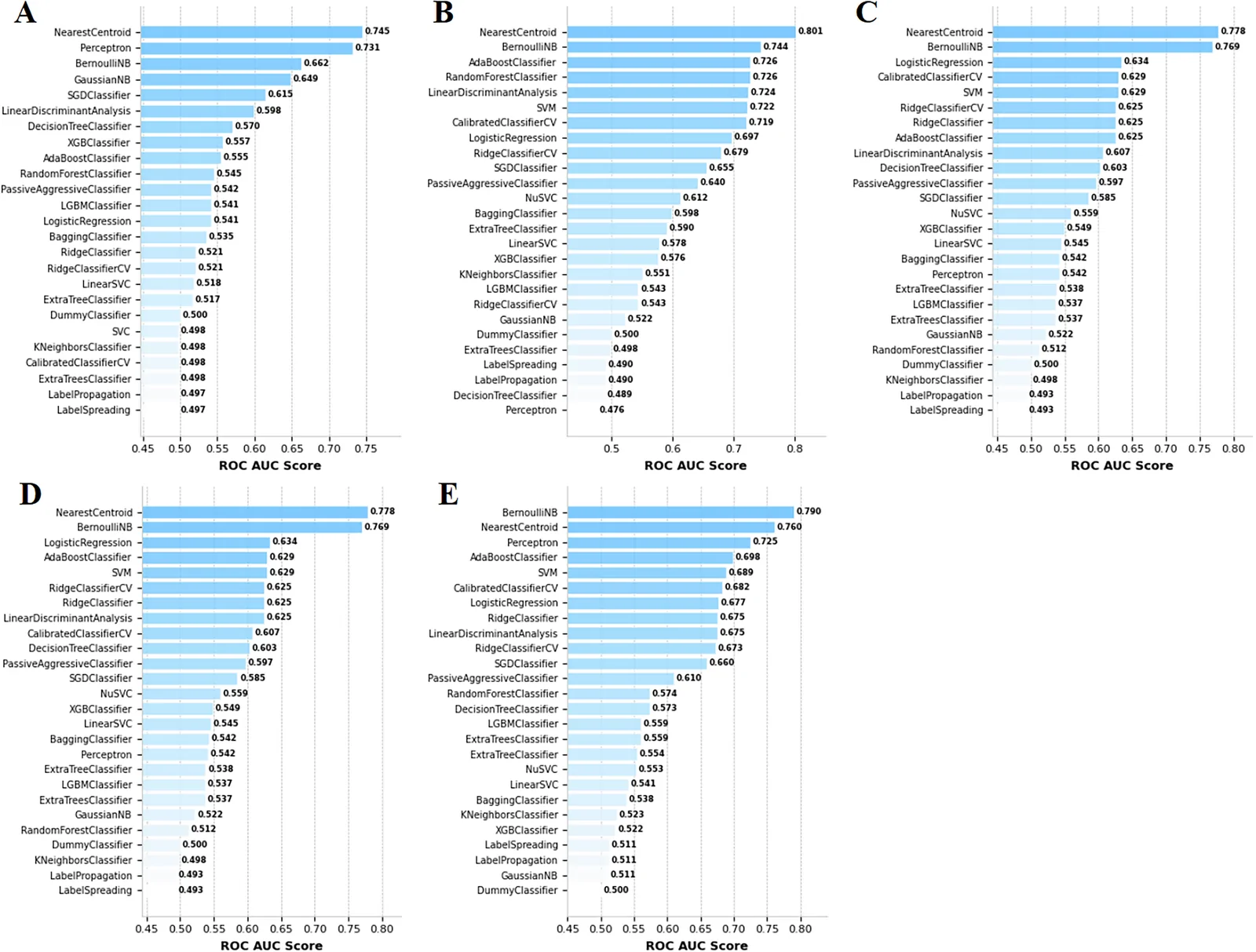
Performance comparison of 26 ML models under five data augmentation strategies. The figure utilizes metrics, primarily the Area Under the Curve (AUC), to evaluate and compare the predictive stability and discriminatory power of multiple algorithms, identifying six top-performing models, including SVM, for further feature refinement. **A** None; **B** random over; **C** smote; **D** smote_tomek; **E** adasyn.

Using clinical data collected within 24 h before surgery, we constructed 26 ML models to predict the occurrence of postoperative LEDVT in patients with EC. As shown in Fig. 2, under five different data augmentation strategies, six models—NearestCentroid, BernoulliNB, RandomForestClassifier, AdaBoostClassifier, SVM, and LogisticRegression—demonstrated stable in terms of AUC. They were therefore included in the subsequent feature selection analysis.

### Feature selection and final model identification

Table 2 presents the importance rankings of the 27 candidate features across the six ML algorithms. The features are listed in descending order of importance as follows: postoperative D-dimer level, age, fibrinogen level, clinical stage, postoperative continuous bed rest time, hematocrit, red blood cell count, operation time, thrombin time (TT), pathological grade, hypertension, postoperative bilateral lower limb massage, lymph node metastasis, activated partial thromboplastin time (APTT), diabetes, vascular invasion, intraoperative fluid infusion volume, maximum tumor diameter, white blood cell count, intraoperative blood loss, platelet count, intraoperative blood transfusion volume, use of hemostatic drugs, menopausal status, body mass index (BMI), use of anticoagulant drugs, and indwelling central venous catheter.

**Table 2:** Model importance ranking based on multiple machine learning algorithms

Variables	Nearest-	Bernoulli	Logistic	Random		Ada-		
	Centroid	NB	regression	Forest	SVM	Boost	average	order
Postoperative D-dimer level	1	5	1	1	4	1	2.167	1
Age	5	13	10	6	7	3	7.333	2
Fibrinogen level	4	8	18	2	6	7	7.500	3
Clinical stage	2	3	8	12	18	12	9.167	4
Postoperative continuous bed rest time	3	7	14	16	8	10	9.667	5
Hematocrit	10	16	6	10	5	16	10.500	6
Total red blood cell count	9	21	20	4	11	2	11.167	7
Operation time,min	11	17	25	5	9	8	12.500	8
Thrombin time	18	22	21	7	1	6	12.500	8
Pathological grade	6	10	11	15	21	14	12.833	10
Hypertension	19	11	7	18	3	19	12.833	10
Postoperative massage of both lower limbs	14	18	3	17	13	13	13.000	12
Lymph node metastasis	23	2	2	25	16	11	13.167	13
Activated partial thromboplastin time	8	24	17	3	27	5	14.000	14
Diabetes mellitus	20	6	4	24	12	20	14.333	15
Vascular invasion	17	4	9	19	14	27	15.000	16
Intraoperative fluid administration volume	15	20	26	11	2	17	15.167	17
Maximum tumor diameter	12	14	13	13	23	18	15.500	18
Total white blood cell count	27	26	15	8	10	9	15.833	19
Intraoperative blood loss	13	12	16	14	26	21	17.000	20
Total platelet count	21	27	27	9	15	4	17.167	21
Intraoperative blood transfusion volume	7	9	24	21	25	25	18.500	22
Hemostatic agent administration	26	23	5	23	22	15	19.000	23
Menopausal status	16	15	22	22	17	24	19.333	24
BMI	25	1	23	27	20	26	20.333	25
Anticoagulant administration	22	19	19	20	24	22	21.000	26
Central venous catheter placement	24	25	12	26	19	23	21.500	27

The table shows the ranking of variables by different models and their average ranking.

**Fig. 3:**
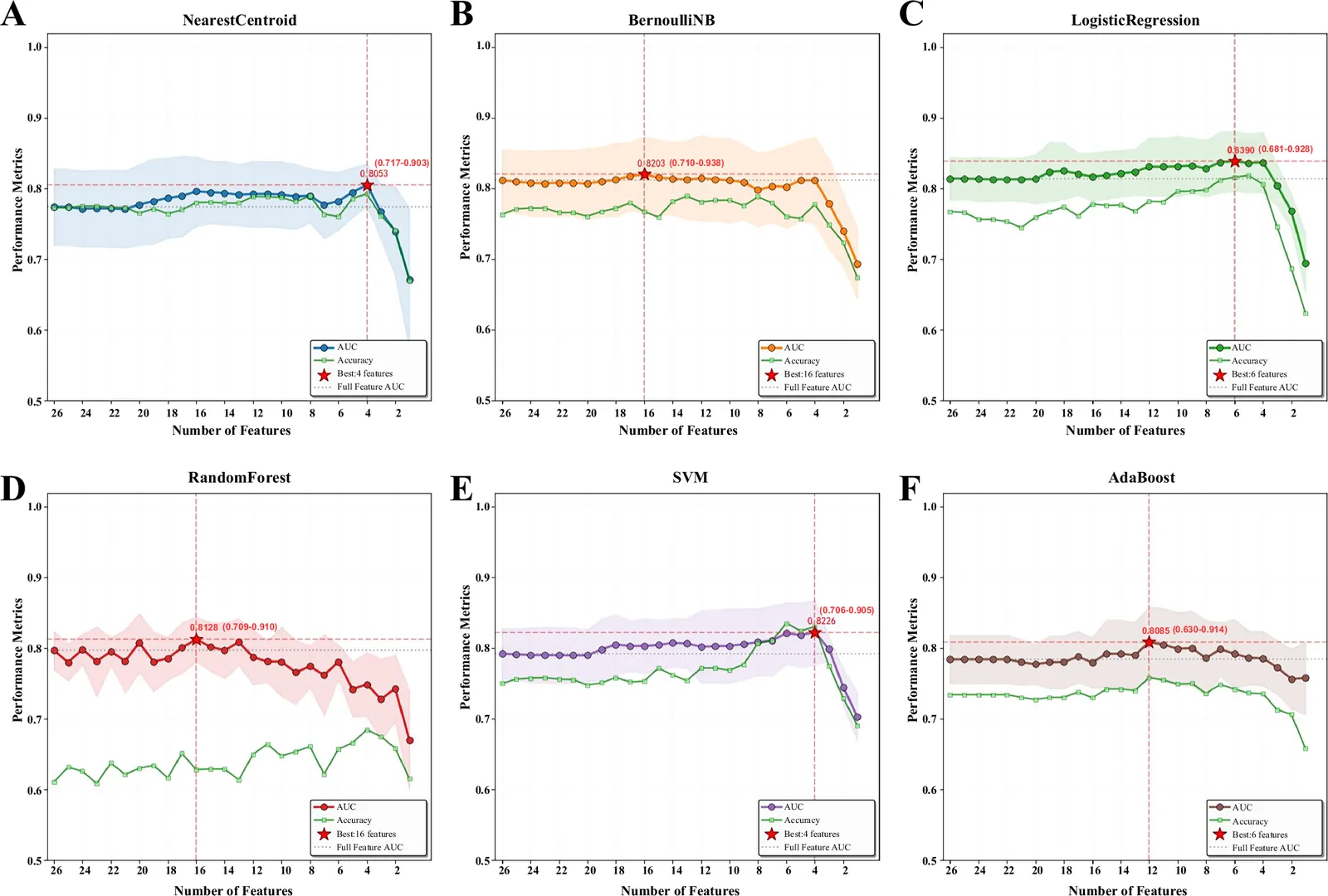
Feature selection analysis via Recursive Feature Elimination (RFE). The plot illustrates the impact of feature reduction on model performance, demonstrating that the SVM model maintains high predictive accuracy (*AUC* ≈ 0.82) even when streamlined to just four essential clinical variables. **A** Nearest Centroid; **B** BernoulliNB; **C** Logistic Regression; **D** Random Forest; **E** SVM; **F** AdaBoost.

As shown in Fig. 3, recursive feature elimination (RFE) analysis indicated that most models maintained relatively high performance when the number of features was reduced to below 10. Among them, the SVM model achieved performance close to that of the full feature set (AUC = 0.82) using only four features, demonstrating strong feature selection capability.

Based on the multi-model RFE results and clinical consensus, the following four core variables were ultimately selected for the final model (SVM): postoperative D-dimer level, age, fibrinogen level, and clinical stage.

### Internal and external validation of the final model

**Fig. 4:**
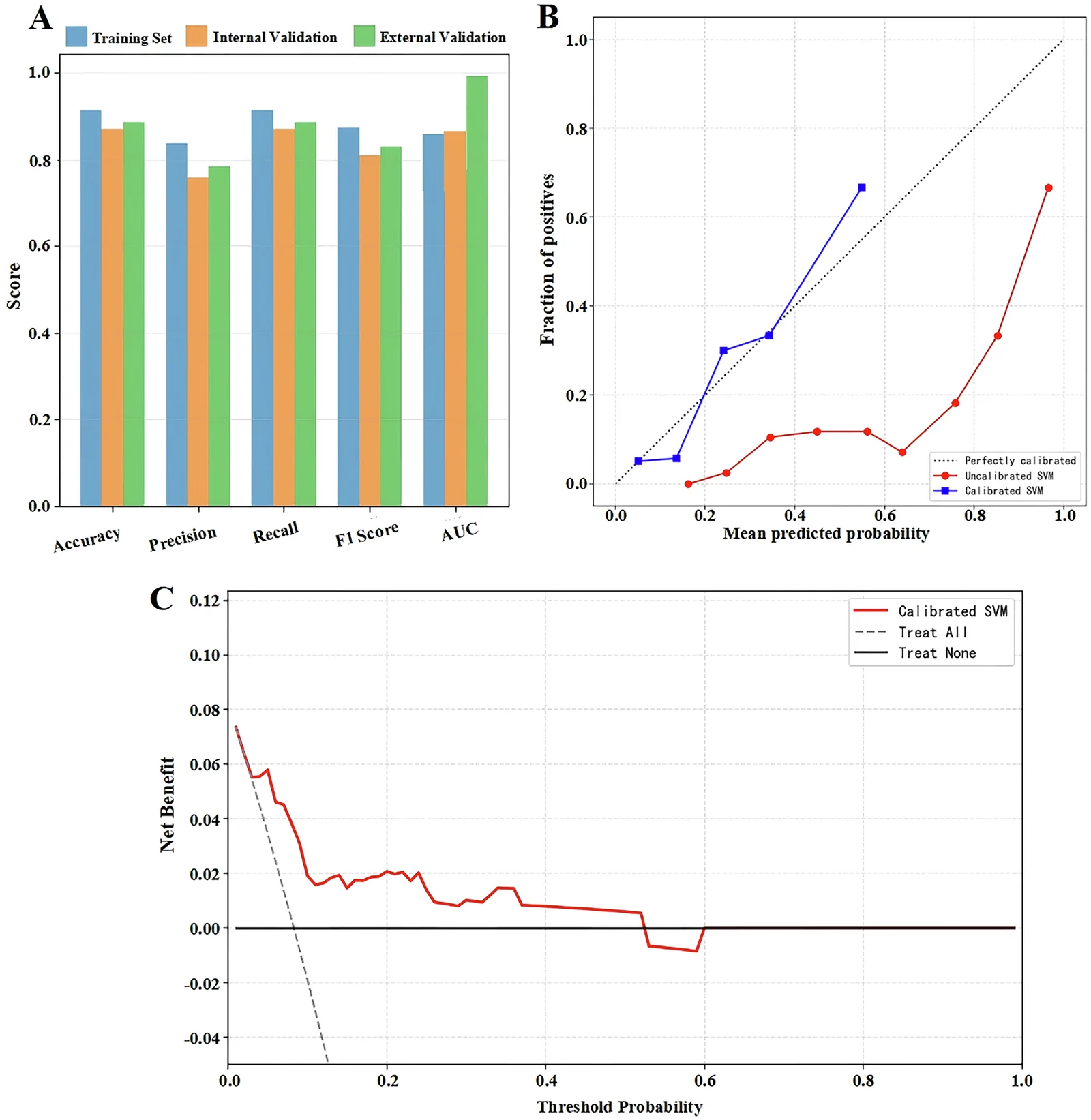
Performance of the final SVM model. The figure presents Receiver Operating Characteristic (ROC) curves and calibration plots across the training, internal, and independent external validation sets, confirming the model’s robust discriminative performance and clinical reliability. **A** Internal and external validation performance. **B** Calibration curves. **C** Decision curve analysis (DCA).

Figure 4 presents the internal and external validation results of the final model. The constructed model demonstrated good predictive performance and generalization ability. On the training set, the model achieved excellent discrimination (AUC = 0.823). In the internal validation set, the model performance remained stable (AUC = 0.828 95% CI:[0.706–0.905]), indicating no significant overfitting. On the independent external validation set, the model yielded an AUC of 0.819, which is highly consistent with the internal validation performance, further confirming the model’s good generalization capability. Furthermore, the model demonstrated good calibration in both the internal and external validation sets, as evidenced by the calibration plot (Fig. 4B).

### Model explanation

**Fig. 5:**
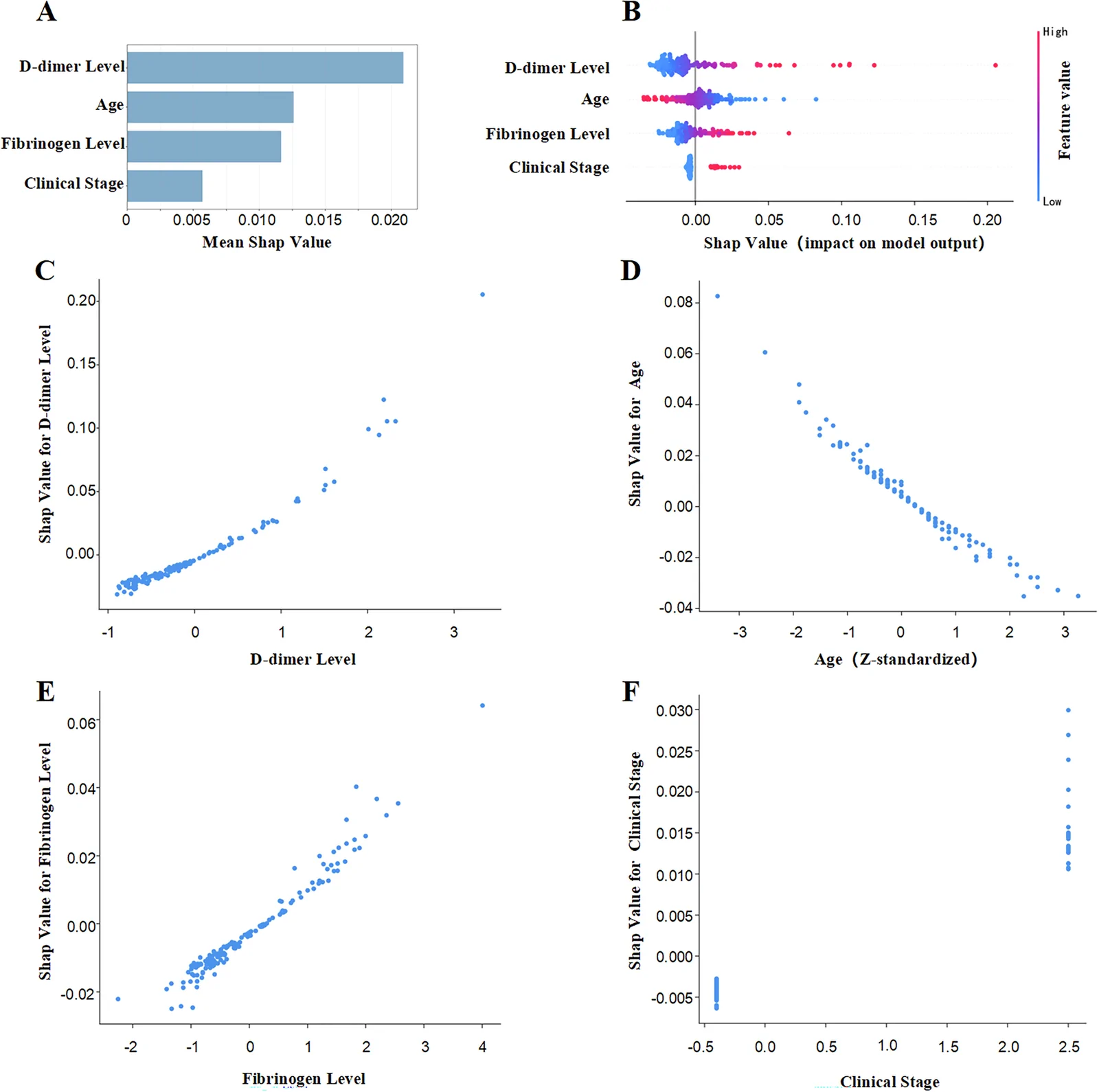
Global feature importance and non-linear dependency analysis based on SHAP values. SHAP summary and dependence plots are employed to quantify the influence of the four key predictors—postoperative D-dimer, age, fibrinogen, and clinical stage—and to visualize how their varying values contribute to the predicted probability of LEDVT. **A** Bar plot of SHAP summary; **B** Dot plot of SHAP summary; **C** SHAP dependence plot of D-dimer level; **D** SHAP dependence plot of age; **E** SHAP dependence plot of fibrinogen level; **F** SHAP dependence plot of clinical stage.

At the global feature importance level, we used the mean absolute SHAP value to assess the contribution magnitude and direction of each feature to the model’s predictions, displayed in descending order. As shown in the SHAP summary plots (Fig. 5A, B), postoperative D-dimer level (mean ∣SHAP∣ = 0.06), age (0.05), fibrinogen level (0.02), and clinical stage (0.01) were the four most influential features affecting the model’s predictions. Furthermore, we analyzed the nonlinear relationships between each feature and the predicted risk using SHAP dependence plots. A SHAP value greater than zero indicates that the feature, at its current value, contributes positively to pushing the prediction towards the “LEDVT" class, suggesting an increased risk of postoperative LEDVT. As shown in Fig. 5C, the postoperative D-dimer level exhibited a clear positive correlation with SHAP values. When its standardized value increased from ~−1 to 1, the corresponding SHAP value rose from about −0.05 to 0.05, indicating that higher D-dimer levels are associated with an increased risk of LEDVT. Figure 5D shows that age demonstrated a “U-shaped" association. The SHAP values were close to zero within the mid-range but decreased to ~−0.04 or increased to about 0.08 at the distribution extremes (particularly on the higher age side), suggesting altered LEDVT risk in patients with extreme ages. As illustrated in Fig. 5E, fibrinogen levels within the lower range (standardized value <0) mostly corresponded to negative SHAP values, hinting at a potential protective effect. However, when the standardized value exceeded ~2.0, the SHAP values tended to become positive, indicating that high levels become a risk factor. Figure 5F shows that the SHAP value increased with advancing clinical stage. Especially after the standardized value exceeded about 1.5, the SHAP value could rise to around 0.03, demonstrating that the degree of disease progression is closely related to the risk of postoperative LEDVT.

**Fig. 6:**
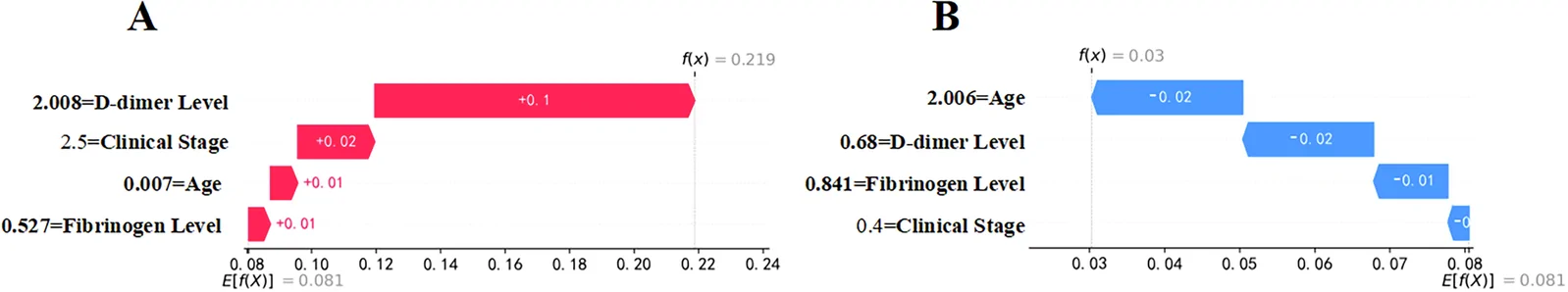
Local interpretation of individualized risk predictions using SHAP waterfall plots. Waterfall plot and evolution of risks contributed by each feature for individual papient at high (**A**) or low (**B**) risk of LEDVT.

At the individual level, SHAP waterfall plots (Fig. 6A, B) visually reveal the decision logic of the model in providing personalized risk predictions based on each patient’s unique combination of clinical features. Figure 6A (high-risk patient) illustrates the model’s assessment pathway for a patient who truly experienced thrombosis. The model output value for this patient (f(x) = 0.219) is significantly higher than the average baseline level of all patients (E[f(X)] = 0.081). The decision process shows that an elevated postoperative D-dimer level (2.008) is the most important positive driver of this prediction, contributing a SHAP value of +0.1. At the same time, an advanced clinical stage (2.5) provides an additional positive contribution (+0.02). Although the patient is relatively young (0.007) and has only a mildly elevated fibrinogen level (0.527), each contributing +0.01, the combination of D-dimer and clinical stage—two strong risk signals—is sufficient to raise the model output from the baseline to 0.219, accurately identifying the high-risk status. In sharp contrast, Fig. 6B (low-risk patient) shows a patient who did not develop thrombosis, with a model output value (f(x) = 0.03) far below the average patient baseline (E[f(X)] = 0.081). The decision path here follows a distinctly different pattern: although the patient’s older age (2.006) contributes a slight positive influence (+0.02), the lower postoperative D-dimer level (0.68) acts as the most critical protective factor, contributing the largest negative SHAP value (−0.02). Furthermore, an earlier clinical stage (0.4) and a non-significantly elevated fibrinogen level (0.841) also provide notable negative contributions (~−0.04 from clinical stage and −0.01 from fibrinogen). The combined effect of these protective features, especially the strong negative influences of clinical stage and D-dimer, pulls the model output significantly down from the baseline to 0.03, resulting in a low-risk judgment consistent with the clinical outcome.

### Clinical application

**Fig. 7:**
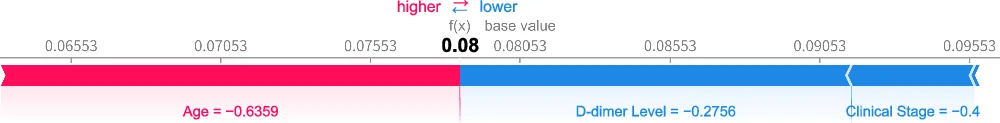
Convenient application for clinical utility. The convenient application of the final SVM model with 4 features is available for LEDVT prediction. When entering actual values of the 4 features, this application automatically displays the probability of 8.5%. Meanwhile, the force plot for individual patient indicates the features that contribute to the decision of “LEDVT'': the blue features on the right are the features pushing the prediction towards the “non-LEDVT” class, while the red features on the left are pushing the prediction towards the “LEDVT” class.

To promote clinical translation and application, this study developed a prototype of a visual decision interface based on the final model. After clinicians input the actual values of the four perioperative features, the system can immediately output an individualized DVT probability and simultaneously generate a force plot for interpretation. As shown in Fig. 7, the predicted probability calculated by the system for a certain patient is 0.085. The SHAP force plot visually demonstrates that younger age, lower postoperative D-dimer levels, and an early clinical stage together constitute the main risk-reducing factors. Their combined effect shifts the predicted value from the baseline level (shown on the right) toward the “lower" direction, resulting in the current risk assessment outcome.

Furthermore, the Decision Curve Analysis (DCA) results demonstrated that using the calibrated SVM model provided a positive net clinical benefit across a risk threshold range of 5–52% (Fig. 4B). In clinical translation, clinicians can flexibly set the decision threshold *P*_*t*_ within this effective range based on the magnitude of side effects associated with specific interventions. When a patient’s individual predicted probability of thrombosis (P) output by the model is *P* ≥ *P*_*t*_, initiating the corresponding intervention is recommended; conversely, when *P* < *P*_*t*_, withholding high-risk interventions is advised to avoid harm from overtreatment. Compared to a “one-size-fits-all" strategy (e.g., treat-all or treat-none), this model-based individualized decision-making can significantly improve overall patient prognosis.

## Discussion

This study systematically applied the SHAP interpretable artificial intelligence framework to the prediction of LEDVT following surgery for EC^13^. The individualized prediction model we developed demonstrated excellent performance in both internal and external validation^15^, and its transparent decision-making logic offered a novel approach to achieving precise prevention^16^.

The four core predictors selected by the final model–postoperative D-dimer level, age, fibrinogen level, and clinical stage–all have a clear pathophysiological basis. The postoperative D-dimer level ranked highest in importance, directly reflecting the degree of postoperative fibrinolysis system activation and hypercoagulable state, and serving as a sensitive biomarker for thrombosis formation^17,18^. The “U-shaped" risk association observed for age suggests distinct thrombotic mechanisms in younger patients (potentially related to aggressive tumor biology) and older patients (often accompanied by vascular endothelial dysfunction and reduced mobility), which provides insight for developing age-stratified prevention strategies^19,20^. The nonlinear relationship between fibrinogen and risk indicates that clinical evaluation should focus on the “inflection point" of its abnormal elevation rather than a simple linear correlation^21,22^. Clinical stage comprehensively reflects the systemic impact of tumor burden on the body’s coagulation-inflammation network^23,24^. Compared to traditional static scoring tools, this model captured the complex interactions among these factors through machine learning algorithms, achieving a paradigm shift from population-based risk stratification to individualized probability prediction^11^.

Notably, our data revealed a significantly higher volume of intraoperative intravenous blood transfusion in the LEDVT group compared to the non-LEDVT group (130.56 vs. 42.33). This discrepancy does not stem from differences in standard surgical protocols, but rather reflects the higher surgical complexity associated with advanced disease. As observed in our cohort, patients in the LEDVT group presented with a higher proportion of advanced clinical stages and lymphovascular space invasion. To ensure oncological safety, these patients often required more extensive and challenging surgical maneuvers, which directly contributed to prolonged operative times and increased intraoperative blood loss, thereby necessitating more transfusions to maintain hemodynamic stability. Furthermore, allogeneic blood transfusion itself is a recognized independent risk factor for venous thromboembolism. The transfusion of blood products can trigger a systemic immuno-inflammatory response and exacerbate a hypercoagulable state, which synergistically contributes to the increased risk of postoperative LEDVT.

The most innovative contribution of this study lay in its successful resolution of the “black box” dilemma of machine learning models through the SHAP framework^13,25^. At the global level, SHAP values objectively quantified the average contribution of each feature to the prediction outcome, enabling physicians to understand the key medical evidence underlying the model’s decisions—a clear contrast to the subjective weighting of traditional scoring scales. At the individual level, the SHAP waterfall plots, as shown in Fig. 6, provided an intuitive explanation for each patient, clearly illustrating how each of their features “pushed” the predicted risk above or “pulled” it below the baseline. This “visual interpretability” fundamentally addressed clinicians’ trust barrier toward AI models^26,27^ and served as the cornerstone for integrating such tools into clinical workflows to support the formulation of individualized prevention strategies^28^. The decision interface prototype developed in this study (Fig. 7) demonstrated the feasibility of this translation ^29^.

Despite the promising results, several limitations of this study should be acknowledged. First, although external validation was performed, the external validation cohort was relatively small (*n* = 95) and geographically limited. This may restrict the model’s generalizability and its ability to perform robustly across more diverse patient populations and different clinical settings. Therefore, large-scale, multi-regional prospective studies are needed to further confirm its clinical utility. Second, due to the retrospective design and reliance on routinely available EMR data,the model did not incorporate potentially important variables such as hereditary thrombophilia^30^, early postoperative mobility, and advanced coagulation diagnostic methods like thromboelastography (TEG)^31^. Although TEG provides a comprehensive assessment of global hemostasis and is highly valuable for thrombosis prediction, it is not yet routinely performed for all early-stage EC patients in our centers. Future prospective studies should consider comparing or integrating our model with these advanced diagnostic tools to further enhance predictive accuracy. Third, although our DCA provided a theoretical threshold range for interventions, we did not prospectively validate these specific risk stratification thresholds in a real-world clinical cohort. Fourth, because postoperative imaging was primarily symptom-triggered rather than routinely performed for all patients, our study may be subject to detection bias. This approach could potentially miss asymptomatic LEDVT cases, leading to an underestimation of the true incidence.

Future research should focus on: (1) conducting prospective interventional studies to rigorously evaluate specific decision thresholds (the 5–52% range identified in our DCA) and verify whether risk stratification based on this model can effectively guide individualized prevention and improve net clinical benefit^32^; (2) developing and deploying user-friendly clinical decision support tools for seamless integration with electronic health record systems^28^; (3) collecting longitudinal time-series data to explore the incorporation of postoperative dynamic monitoring indicators into the model, thereby upgrading the current static prediction to a real-time, continuous risk assessment system; (4) extending the interpretable framework of this study to the prediction of other postoperative complica- tions in gynecological oncology

This study developed and validated an interpretable machine learning model based on SHAP for the individualized prediction of postoperative LEDVT risk in patients with EC. The model demonstrated reliable risk assessment capabilities using only four routine perioperative clinical indicators. More importantly, by providing both global and local explanations through the SHAP framework, this study helps mitigate the “black-box" nature of traditional machine learning models, offering a more transparent and understandable reference for clinicians. This approach highlights the value of combining predictive performance with clinical interpretability, providing a practical decision-support tool to assist in the precise prevention of postoperative complications in gynecological oncology.

## Methods

### Ethical consideration

This study was conducted in accordance with the Declaration of Helsinki and was approved by the Ethics Committees of the following institutions: the Affiliated Hospital of Zunyi Medical University (No. KLLY-2024-212), Guizhou Provincial People’s Hospital (No. 2026-121), Yanhe Tujia Autonomous County People’s Hospital (No. 2025015), the Third Affiliated Hospital of Zunyi Medical University (No. (2025)-1-1050), and Liupanshui Maternal and Child Health Hospital (No. KYL-J2024002). Due to the retrospective nature of this study, the requirement for written informed consent was waived by the aforementioned committees.

### Study population

The study included patients who underwent comprehensive staging surgery for early-stage EC in the Department of Gynecology of the Affiliated Hospital of Zunyi Medical University, Guizhou Provincial People’s Hospital, Yanhe Tujia Autonomous County People’s Hospital, the Third Affiliated Hospital of Zunyi Medical University and Liupanshui Maternal and Child Health Hospital between October 2011 and March 2026. All enrolled patients were diagnosed with EC by postoperative pathological examination and received standard surgical treatment. Exclusion criteria included: preoperative anticoagulant therapy, a history of venous thromboembolism, and coexistence of other malignant tumors^2^. The study strictly adhered to the principles of the Declaration of Helsinki and were approved by the Ethics Committee of the five hospitals mentioned above. Due to the retrospective nature of the analysis, the requirement for informed consent was waived^33^.

### Data collection and processing

This study utilized the electronic medical record system to collect clinical data from patients within 24 h before surgery for feature selection and predictive model construction^34^. The collected data primarily included: individual factors, surgery-related factors, postoperative laboratory indicators, and tumor characteristics. To avoid overfitting and ensure model stability, the model derivation cohort was randomly split into a training set (for model development) and a test set (for internal validation) in an 8:2 ratio^35^. Additionally, a dataset comprising patients from April 2025 to March 2026 was used for the external validation of the model^15^. The inclusion and exclusion criteria for this validation cohort were identical to those of the model development cohort.

To reduce bias introduced by missing data, variables with a missing rate exceeding 25% were first excluded. For the remaining variables, missing values were imputed using the median for continuous variables and the mode for categorical variables^36^. After initially considering ~40 candidate predictors, preliminary screening for multicollinearity was performed: Cramér’s V was calculated for discrete variables and Pearson correlation coefficients were computed for continuous variables^37^. Finally, 27 variables were selected for subsequent modeling, including: age, BMI, hypertension, diabetes, menopausal status, maximum tumor diameter, lymph node metastasis, vascular invasion, pathological grade, clinical stage, operation time, intraoperative blood loss, intraoperative blood transfusion, intraoperative fluid infusion, white blood cell count, red blood cell count, hematocrit, platelet count, activated partial thromboplastin time (APTT), thrombin time (TT), postoperative D-dimer level (measured within 24–48 h after surgery), fibrinogen level, postoperative continuous bed rest time, use of hemostatic drugs, use of anticoagulant drugs, postoperative bilateral lower limb massage, and indwelling central venous catheter.

Given that postoperative LEDVT events accounted for only 8.6% (72/841) of the original dataset, indicating a significant class imbalance problem, five data resampling strategies were employed for data augmentation: ADASYN, none, random over, SMOTE, and SMOTE-Tomek. Based on the mean AUC metric, the optimal data augmentation method was selected as random over. Subsequent ML models were trained on the augmented balanced dataset to ensure the stability and reliability of predictive performance^38^.

### Definition of LEDVT

In this study, “postoperative LEDVT” was defined as newly developed deep venous thrombosis of the lower extremity (including the femoral, popliteal, tibiofibular venous plexus or iliac veins) occurring within 30 days after radical surgery for EC^2,39^. The diagnosis required confirmation by color Doppler ultrasonography or CT venography, with imaging findings meeting at least one of the following criteria: a solid hypoechoic or hypodense filling defect within the venous lumen; complete or partial absence of blood flow signal at the defect site on color Doppler; or non-compressibility of the venous lumen on compression test^40^. The time of diagnosis was based on the date of the first positive imaging report after surgery. Given the retrospective design of this study, imaging examinations were not routinely performed for all patients but were primarily triggered by clinical symptoms or abnormal laboratory findings. Furthermore, the exact timing of imaging examinations was not strictly standardized across centers. Importantly, to ensure the model serves as a true perioperative risk prediction tool rather than reflecting early detection, we verified that the postoperative D-dimer and other laboratory measurements strictly preceded the first diagnostic imaging for LEDVT in all cases. Only cases of new-onset LEDVT after surgery were included, while patients with pre-existing LEDVT or those developing LEDVT more than 30 days after surgery were excluded^2^.

### Model development and comparison

This study incorporated a total of 26 ML algorithms for model construction and performance comparison, including: NearestCentroid, BernoulliNB, RandomForestClassifier, AdaBoostClassifier, SVM, LogisticRegression, LinearDiscriminantAnalysis, CalibratedClassifierCV, RidgeClassifierCV, SGDClassifier, PassiveAggressiveClassifier, NuSVC, BaggingClassifier, ExtraTreeClassifier, LinearSVC, XGBClassifier, KNeighborsClassifier, RidgeClassifierCV, LGBMClassifier, GaussianNB, DummyClassifier, ExtraTreesClassifier, LabelSpreading, LabelPropagation, DecisionTreeClassifier, and Perceptron^41^.

During the training phase, stratified 5-fold cross-validation (CV) was employed for hyperparameter tuning^42^. To rigorously prevent information leakage, the data processing pipeline was structured as follows: initial train-test split → 5-fold CV within the training set → random oversampling applied strictly within the training folds of each CV iteration → model training → evaluation on the untouched validation folds. The internal test set and external validation set were kept completely unseen and untouched by any resampling process. The final parameter combination for each algorithm was determined through a combination of grid search and manual fine-tuning. All models were implemented and evaluated in the same computational environment (Python 3.9/scikit-learn 1.1). The complete source code and the trained model weights are publicly available on GitHub.

Model performance was comprehensively evaluated using the following common metrics: the area under the receiver operating characteristic (ROC) curve (AUC), Accuracy, Precision, Recall, Matthews Correlation Coefficient (MCC), and the F1-score^43,44^. The AUC served as the primary criterion for ranking to select the optimal algorithm for subsequent feature selection and model interpretation. Additionally, to assess the agreement between predicted probabilities and observed outcomes, we evaluated model calibration using calibration plots.

### Feature selection and model interpretation

To address the “black box" limitation of ML, this study introduced the SHAP framework to systematically interpret the optimal model^13^. First, based on the training set, SHAP values were calculated for each sample and ranked in descending order of their mean absolute values to generate a feature importance ranking list. Subsequently, a “recursive elimination" process was implemented: starting with all 27 features, the least important feature was iteratively removed, and the AUC on the internal validation set was recalculated at each step. The elimination process stopped when the AUC decreased by more than 0.02, thereby identifying the key variables and achieving an optimal balance between model performance and simplicity^45^.

To enhance model credibility and meet clinical interpretability needs, the SHAP method was used to interpret the final model’s output by quantifying the contribution of each variable to the prediction^12^. This interpretability approach provides two types of explanations: global interpretation at the feature level and local interpretation at the individual level. Global interpretation aggregates SHAP values across all samples, assigning a consistent and rankable contribution score to each feature, thereby quantifying the overall association strength between input variables and postoperative LEDVT risk. Local interpretation allows the actual variable values of a specific patient to be input into the model, generating a force plot in real time to visually illustrate how each feature contributes to “pushing" the prediction toward or “pulling" it away from the LEDVT outcome for that individual^14^. This enables precise clinical interpretation characterized by “one prediction, one plot, one explanation".

## Data Availability

The datasets generated and/or analyzed during the current study are not publicly available due to patient privacy and institutional data protection policies but are available from the corresponding author on reasonable request. De-identified data can be provided after signing a data use agreement.

## Code availability

The complete Python scripts for model training, feature elimination, and evaluation, along with the trained final model weights used in this study, have been deposited in a public GitHub repository: https://github.com/cyh407/Personalised-thrombo-embolic-risk-prediction-after-endometrial-cancer-surgery.
